# Impact of integrated teaching-learning method on oncology clinical decision-making ability and cognitive learning of nursing students

**DOI:** 10.1186/s12909-022-03168-2

**Published:** 2022-02-19

**Authors:** Arefeh Davoodi, Vahid Zamanzadeh, Akram Ghahramanian, Tonia C. Onyeka, Faranak Jabbarzadeh

**Affiliations:** 1 Educator, Medical Education Research Center, Health Management and Safety Promotion Research Institute, Department of Medical-Surgical Nursing, Faculty of Nursing and Midwifery, Tabriz University of Medical Sciences, Tabriz, Iran; 2 Professor, Department of Medical-Surgical Nursing, Faculty of Nursing and Midwifery, Tabriz University of Medical Sciences, Tabriz, Iran; 3 Associate Professor, Medical Education Research Center, Health Management and Safety Promotion Research Institute, Department of Medical-Surgical Nursing, Faculty of Nursing and Midwifery, , Tabriz University of Medical Sciences, Tabriz, Iran; 4 Department of Anesthesia/Pain & Palliative Care Unit, College of Medicine, University of Nigeria, Ituku-Ozalla Campus, Enugu State Nsukka, Nigeria

**Keywords:** Integrated teaching-learning, Clinical Education, Nursing, Clinical decision-making, Cognitive learning, Internship

## Abstract

**Background:**

Innovative and student-centered teaching methods are required to improve critical thinking and clinical reasoning skills. The objective of this study was to determine the impact of an oncology internship training on learning outcomes of nursing students using an integrated teaching-learning method.

**Methods:**

A pre- and post-test quasi-experimental study was conducted among 107 undergraduate nursing students in fourth year who were allocated to two groups (intervention group = 51 and control group = 55) to receive an integrated teaching-learning method and routine method respectively. Data was collected using the Clinical Decision Making in Nursing Scale (CDMNS) and the students’ cognitive learning test.

**Results:**

Difference in mean scores of cognitive learning test post-intervention was significant between the two groups (*p* < 0.001). Total CDMNS scores and its dimensions increased significantly for the intervention group post-intervention (*p* < 0.001). Analysis of covariance **(**ANCOVA) showed that when the effect of confounding variables, such as the student’s Grade Point Average (GPA) and the pre-test scores of cognitive learning and decision-making scale were held constant, the effect of the independent variable (group) on students’ cognitive learning test (*p* = 0.002) and CDMNS (*p* = 0.004) was significant.

**Conclusions:**

Nursing students’ cognitive learning and clinical decision-making scores were improved as a result of the integrated teaching-learning method. Nursing educators can use this method in clinical education to improve students’ cognitive and meta-cognitive skills, thereby improving nursing care quality.

**Supplementary Information:**

The online version contains supplementary material available at 10.1186/s12909-022-03168-2.

## Background

One of the major issues in clinical education today is the gap between theory and practice [1]. Furthermore, from the perspective of educators and students, clinical education programs which are offered in internships are of medium quality and do not develop the necessary capabilities in cognitive, emotional and psychological aspects of the student’s abilities to the desired level [2]. Students are expected to apply classroom science to clinical areas and use the best available evidence to improve clinical decision-making and cognitive skills in the clinics and the wards [3]. To achieve this, nursing educators have been known to use creative and student-centered teaching methods to promote cognitive skills and application of theory in clinical duties in nursing education. Some of these methods include concept map [4], lectures, role-playing, clinical journal clubs, clinical conferences, clinical reports, and case presentations [5]. Also, collaborative learning employed in clinical education has been found useful for improving student clinical competency and skills, strengthening teamwork as well as promoting motivation and self-confidence [6]. Previous research have shown that using integrated teaching-learning methods leads to increased competence and clinical qualification of nurses [7], by promoting a shift from theoretical knowledge to effective and conscious performance [8], strengthening of data collection and analysis process, increasing the ability to prioritize problems to achieve ideal solutions [9], all leading to improved practical and theoretical nursing student grades [10]. The use of an integrated method of case study and nursing process strengthens students’ reflection even further, because students are able to obtain more information about their patients, which when combined with other information obtained during clinical examination of patients, leads to a more accurate diagnosis of the patient’s needs and problems, and better care measures [2]. From experience, it is observed that integrated education is one of the useful methods that improves the quality of education in the clinic and produces more effective learning outcomes than conventional education.

Several barriers to the successful clinical education of Iranian nursing students exist [11]. These include the barriers of clinical environment such as lack of equipment and appropriate facilities for learning [12–14]. In addition, there is a lack of qualified instructors and a lack of variation in teaching and learning strategies [11]. With regards to the latter, the quality of clinical education has decreased, and practical training is not suitable for developing students’ critical thinking and clinical decision-making skills, as recognized by many nursing students and educators [13]. Thus, in order to address this serious issue which ultimately poses a threat to community health, given that the health transformation plan in medical education emphasizes the training of efficient manpower and the improvement of community health, empowering human resources in nursing is an important step toward achieving this goal. Therefore, the purpose of this study was to determine how an integrated teaching-learning method comprising Nursing Process and Case Study would affect the cognitive learning and clinical decision-making capacities of undergraduate nursing students.

## Methods

### Design and setting

The design used in this quantitative research was a quasi-experimental approach with a pre- and post-design. In Iran, a bachelor’s degree in nursing is 4 years. Students complete two semesters each year and complete 130 credits over four years. Each theoretical, practical and workshop / internship unit is equivalent to 17, 34 and 51 h respectively. Students in the first semester of the fourth year undertake the Adult and Elderly Nursing Internship course (8 credits) and the Cancer Nursing Internship course, both scheduled for a total of 6 days (0.5 credits). In Tabriz School of Nursing and Midwifery, the oncology nursing internship program is planned for students in the 7th semester. Research setting was the Shahid Ghazi Hospital, where is affiliated to Tabriz University of Medical Sciences. The first author of this article developed the intervention protocol in collaboration with the research team. The protocol was implemented during the cancer nursing internship for undergraduate nursing students in 2019-2020 by first author.

### Participants

**Fig. 1:**
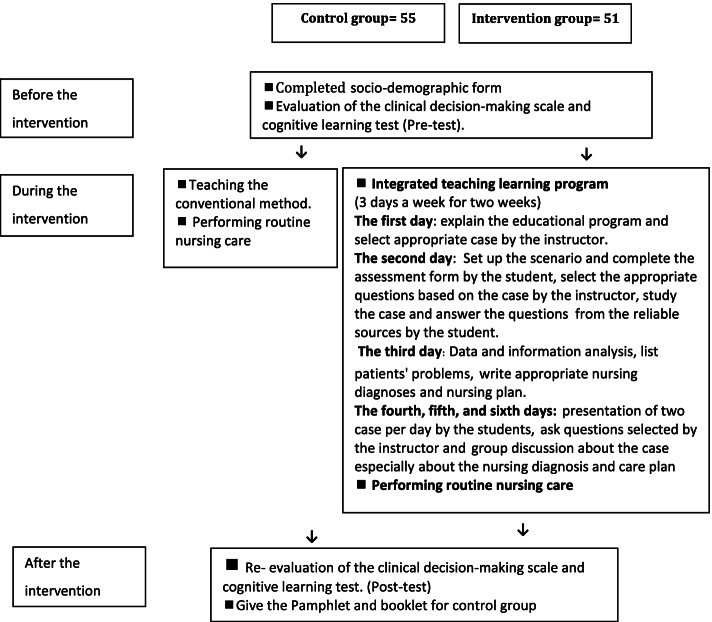
Overview of the research protocol. Integrated teaching learning program

In this research, 107 undergraduate nursing students in the 7th semester formed the research population. A total of 55 students between September 2019 and December 2019, who entered in this internship program, formed the control group, and another 51 students, who entered this program between January 2020 and May 2020, were selected for the intervention group. The reason for this choice was to prevent information dissemination of the intervention from intervention group to control group and to be able to collect the control group data before the intervention group (Fig. 1).

Participants in the intervention group were taught using the integrated teaching-learning method, while the control group was taught using the conventional method. In the conventional method, students assessed their patients after assigning 1-2 patients to them and took care of their patients under the supervision of an instructor based on their nursing diagnoses and patient care plan. Participants in both groups were comparable in terms of demographic characteristics after being assigned. Students were included in the study if they had passed a theoretical nursing course for Hematology and Oncology diseases during the Adult and Elderly Nursing-3 course unit. Inability to complete the courses and absence from the wards for a minimum of one day served as the exclusion criteria.

### Data collection tools

Data collection tools included a socio-demographic form, a clinical decision-making scale and a cognitive learning test that students completed before and after the training. Age, gender, marital status, nursing experience, and GPA were among the socio-demographic variables obtained.

The Clinical Decision Making in Nursing Scale (CDMNS) was developed by Jenkins (1985). It assesses nurses’ clinical decision-making skills and has four subscales: (1) Search for alternatives or options (2) Canvassing of objectives and values (3) Evaluation and re-evaluation of consequences (4) Search for information and unbiased assimilation of new information. It includes 40 items to which the participants’ responses (which are based on their understanding of the clinical decision-making situation) are rated on a Likert scale as follows: 5 - always; 4 - most of the time; 3 - occasionally; 2 - rarely; 1 - never; 0. Range of scores are between 40 and 200 with higher scores indicating higher clinical decision-making capabilities. This tool has been found to have favorable internal consistency with a Cronbach’s alpha of 0.83 [15]. Lotfi et al. have previously confirmed the scientific validity of this scale in a study aimed at determining the effect of integrated simulation training and critical thinking strategies on nurses’ clinical decision-making skill [16]. The validity and reliability of CDMNS was also confirmed in this present study after translation by three bilingual nursing specialists using the Forward method and following testing on a group of ten nursing students who were not enrolled into this present study, using the face validity method and the scale was found to have a Cronbach’s alpha value of 0.89, indicating a good internal consistency.

The cognitive learning test which was a teacher-made test, was developed for the study. Teacher-made test is an important tool used by teachers to appraise the teaching method of the class for which it is prepared [17]. The test in this study consisted of 25 multiple-choice questions designed to determine the degree to which course objectives were met. Each question has a stem with four response options (distractors) from which the respondent is required to select the correct option/best one. Based on the course plan, course content, and objectives, the test was designed to assess high levels of cognitive learning. Each correct option received a score of one and the possible range of scores was between 0 and 25. A Table of Specification (TOS) approach was used to ensure that the content was relevant and inclusive. A TOS is defined as a test blueprint which helps teachers to align objectives, instruction, activity and assessment [18, 19]. The reliability coefficient using the Kuder-Richardson-21 formula was calculated to be 0.92.

### Statistical data analysis

Following data collection and coding, analysis was conducted using SPSS Statistics 16.0 (2008). Independent t-test and paired t-test were used to compare mean scores of the intervention and control groups in the cognitive learning test and the CDMNS before and after the integrated teaching-learning method. Comparison of baseline parameters was performed with independent t-test and Analysis of variance. Given that the intervention and control groups had significant differences in GPA prior to the intervention, and the intervention group had a significantly higher GPA, analysis of covariance (ANCOVA) was employed.

### The intervention protocol

The first author of article, who was also the internship course instructor, explained the integrated teaching-learning method to the intervention group students and guided the students during the course. The intervention groups were divided into 9 groups. Each internship course lasted two weeks (three morning shifts per week) and were attended by six to seven students in each group. On the first day of the internship common hematology and oncology diseases were selected as the case for example: Acute myeloid and lymphoid leukemia, Hodgkin’s and non-Hodgkin’s lymphoma, Multiple Myeloma, Aplastic Anemia, and other bleeding disorders. Given that each student was introduced to one case of hematology and oncology disease, the group as a whole was exposed to 6 to 7 different hematology and oncology cases.

 Along with routine nursing care, the students began their case study on the second day of the internship. The students required basic knowledge and prior experience to do the case study and set up the scenario. The students had completed 5 theoretical credits regarding blood and cancer during the semester, and on the first day of the internship, they were told to study their cases and be ready to assessment patients. Data about each case was collected by the students using a health status survey form designed by the first author and based on reputable books of oncology nursing. On the second day of the internship, students completed two critical tasks: creating a case study in the form of a scenario and completing the first stage of the nursing process (assessment and developing an information database). It took 2 h to assess, recognize, and set up the scenario. This was a critical step that had to be completed correctly. To this end, the instructor guided the students through the case study and ensured that the students obtained valuable information from the patient.

The material written in the scenario was regarded as a learning tool, and some additional essential information was required, which the students had to acquire in order to learn more about the case. For this purpose, after the student created the scenario, the instructor reviewed it and created appropriate questions about the patient that were tailored to the student’s learning needs. For example, if a bone marrow biopsy was performed on a patient, the instructor would inquire about pre- and post-biopsy nursing care, or if the patient’s treatment regimen included Taxoter and Cisplatin, the instructor would inquire about the regimen, as well as its medication and side effects. The content of the case study questions came from the patients themselves, and the case was viewed as a clue to the students’ learning. After designing useful questions, the instructor would guide the students, so that they could get answers to the questions and complete information about the case. The learning was exploratory in nature.

On the third day of the internship, students were required to summarize their patient’s situation and with guidance from the instructor, describe the illness, present assessment findings, develop nursing diagnoses and a holistic plan of care. The cases were then presented to the rest of the clinical group. So that on the second week of the internship, two of the case studies were presented for about 15 to 20 min by the students. Selected questions about the case were asked for 30 min, and other students in the group expressed their opinions. The nursing diagnoses and care plan were then discussed in the group, and the instructor finally provided the necessary feedback in achieving the learning goals and care plan. Thus, during the second three days, all of the students in the group received good information about 6 to 7 different cases and became acquainted with these cases which they would most likely face in their future nursing career and would provide quality and safe nursing care.

It should be noted that during the six days of the internship, cognitive skills were taught alongside clinical skills. In addition to clinical care, the students improved in their cognitive abilities. The instructor attempted, as much as possible, to have students take care of the patients and to observe, step by step, the patient’s state of progress and recovery, as well as the state of treatment, so that a meaningful, deep, and sustainable learning was created. A summary of the intervention protocol for the intervention group is provided in Additional file 1.

## Results

The results of the independent t-test revealed that there was no difference between the control and intervention groups in terms of age, work experience, marital status, or gender, but there was a statistically significant difference in GPA, with the intervention group having a higher GPA than the control group (Table 1).

**Table 1:** Comparison of baseline parameters

Characteristics	Intervention group (*n *= 51)	Control group (*n *= 55)	*p-value*
Gender			0.56
Female	26 (51)	24(43.6)	
Male	25 (49)	31(56.4)	
marital status			*1*
Married	14 (27.5)	14 (26.4)	
Single	37 (72.5)	39 (73.6)	
Age (Yrs.)	23.55 ± 1.96	22.91 ± 1.42	0.06
Work experience (Yrs.)	1.82 ± 1.64	1.83 ± 1.34	0.96
Grade point average (GPA)	16.40 ± 1.04	15.95 ± 1.16	0.044

In comparing the clinical decision scores and their dimensions between the groups, Table 2 shows that the intervention group had significantly higher total clinical decision-making pre-test scores and dimensions, especially the dimensions of canvassing of objectives and values and goals and evaluation and re-evaluation of consequences. Regarding the post-test scores, a comparison between the groups revealed that the intervention group performed significantly better in the total scores (*p *< 0.001) and in the four dimensions of clinical decision-making (*p *= 0.001).

**Table 2:** Comparison of pre-test and post-test scores of Clinical decision making and cognitive learning between groups

Learning outcomes	Time	Intervention group (*n* = 51)	Control group (*n* = 55)	*p-value®*
**Clinical decision making and its dimensions**				
Total score of Clinical decision making	Pre-test	156.13 ± 12.93	149.14 ± 15.18	0.012
	Post-test	163.98 ± 12.75	151.14 ± 18.60	< 0.001
	*p-value©*	< 0.001	0.399	
Subscale1. Search for alternatives or options	Pre-test	43 ± 4.60	41.32 ± 5.33	0.088
	Post-test	45.49 ± 3.93	42.12 ± 5.52	0.001
	*p-value©*	< 0.001	0.246	
Subscale2. Canvassing of objectives and values	Pre-test	56.75 ± 4.68	53.70 ± 4.97	0.002
	Post-test	57.88 ± 4.82	53.58 ± 7.33	0.001
	*p-value©*	0.069	0.907	
Subscale3. Evaluation and reevaluation of consequences	Pre-test	38.37 ± 3.86	36.45 ± 4.70	0.024
	Post-test	41.01 ± 4.09	37.56 ± 5.64	0.001
	*p-value©*	< 0.001	0.119	
Subscale4. Search for information and unbiased assimilation of new information.	Pre-test	21.74 ± 2.88	21.34 ± 3.60	0.53
	Post-test	23.43 ± 2.80	21.50± 3.06	0.001
	*p-value©*	<0.001	0.730	
**Cognitive learning**				
Total score of cognitive learning test	Pre-test	10.29 ± 3.02	10.56 ± 2.91	0.642
	Post-test	15.17 ± 2.05	13.27 ± 3.01	p < 0.001
	*p-value©*	< 0.001	< 0.001	

*©p-value* for paired t-test; ***®****p-value* for independent t-test

The results of the paired t-test for within-group comparison of pre- and post-test clinical decision-making scores revealed that in the control group, there was no significant increase in post-test scores for total and clinical decision-making dimensions (*p* > 0.05). Whereas in the intervention group, post-test scores were significantly increased in the total score and all dimensions of clinical decision-making (*p *< 0.001), except for the dimension of canvassing of objectives and values (Table 2).

Given that the intervention and control groups had significant differences in GPA prior to the intervention, and the intervention group had a significantly higher GPA, so the analysis of covariance (ANCOVA) was employed. Table 3 show that the intervention had a significant effect on students’ clinical decision-making scores (*p* = 0.004).

**Table 3:** Effect of integrated teaching-learning method on Clinical decision making scores

Parameter	B	*p-value*	95% of CI	Effect size (Eta)
Constant(Fixed amount)	71.826	0.013	15.70-127.94	0.063
Pre-test score	0.594	p < 0.001	0.39-0.79	0.257
Grade point average	-0.029	0.982	-2.60-2.54	*0*
control group (Conventional training)	-8.922	0.004	-14.86—2.98	*0.085*
Intervention group (referent)	-			

The difference in post-test cognitive learning scores between the intervention and control groups was significant, indicating that after integrated teaching-learning program, students’ cognitive learning was higher than the control group (Table 2). The paired t-test results for within-group comparison of cognitive learning scores in the control and intervention groups revealed a significant difference (*p *< 0.001).

Given that the two groups had significant differences in total GPA with the intervention group having a significantly higher GPA, the Analysis of Covariance (ANCOVA) was used in the next stage of the analysis. In this analysis, the mean post-test scores were used as a dependent variable, the group as an independent variable, the GPA, and the mean pre-test cognitive learning scores as confounding variables. The analysis results in Table 4 show that the intervention had a significant effect on students’ cognitive learning scores (*p* = 0.002).

**Table 4:** Effect of integrated teaching-learning method on cognitive learning scores

Parameter	B	*p-value*	95% of CI	Effect size (Eta)
Constant(Fixed amount)	4.19	0.225	-2.63- 11.02	0.015
Pre-test score	0.269	0.002	0.104-0.434	*0.099*
Grade point average	0.499	0.023	0.069-0.930	*0.052*
control group (Conventional training)	-1.520	0.002	-2.461--0.580	*0.097*
Intervention group (referent)	-			

## Discussion

Our hypothesis about the effect of the integrated teaching-learning method on cognitive learning and clinical decision-making ability of students is confirmed by the findings of this study. Learning in this study was exploratory in nature, and the instructor sought to enhance students’ creative and participatory learning as adult learners. In the present study, skills and procedures were taught with the active participation of learners. In addition to performing their clinical duties, the students thoroughly reviewed the clinical cases and developed a care plan for them by going through the steps of the nursing process, and this integrated teaching method kept them active throughout the internship. The findings of other studies indicate that collaboration between teachers and students is necessary and cannot be separated from nursing clinical education. In fact, clinical education should be student-centered, and cooperation between students and teachers should be an essential part of clinical education [6, 20]. The use of problem-based educational strategies prevents nursing students from experiencing classroom teaching and clinical practice as separate Sect. [21]. Tseng (combining Problem-based learning method and concept mapping) [22] and Wang (combining Problem-based learning method and nursing process) [23] conducted two studies in Taiwan, and the results showed that students in the intervention group improved on their cognitive and clinical reasoning skills, as well as their problem-solving ability. The results of another study which used a combination of theoretical and practical teaching methods in CPR learning in China, revealed that the intervention group’s theoretical and practical scores increased, with most students agreeing that the teaching method was effective in clinical problem-solving [15].

In the control group, post-test scores did not increase significantly compared to pre-test scores but in the intervention group, the scores showed a significant increase, except for the dimension of canvassing of objectives and values. The results of the Wang study, which combined the effects of medical science education and nursing care with the simulation method, revealed that nurses’ scores improved in three dimensions: clinical competence, interpersonal relationships, and law and professional ethics [7]. A similar study found that integrating simulation and interpretative pedagogy in a bid to increase students’ ability and clinical competence, led to a shift from theoretical knowledge to effective and conscious clinical practice for the students [8]. Integrated teaching-learning method help to bridge the gap between theory and practice and improve nurses’ clinical competence [24]. After integrated training, medical students’ clinical decision-making ability, as well as midwifery and nursing students’ clinical reasoning have been known to significantly improve [25–27]. In addition, the integrated teaching-learning method has been shown to increase satisfaction and to result in high self-esteem in nursing students [28].

In this study, the cognitive skills of the students improved in the areas of critical thinking and clinical reasoning, and the link was established between theory and clinical practice. The ability to assess and recognize the patient, review different body systems and interpret relevant blood tests, in addition to the information contained in the scenario narrative, improved the students’ understanding of the patient’s clinical problems and equally improved their ability to delineate the latter from other issues related to the psychological, mental, emotional and spiritual dimension. This has the potential to help such students develop and implement a care plan when faced with similar clinical situations in future. A careful examination of a case scenario sharpened the focus on one patient, resulting in an improvement in the patient’s clinical condition. Because the students’ internship time was managed and the students were involved in ward tasks and the opportunities were used well, the implementation of this educational method led to increased self-confidence, interest, motivation, and satisfaction in students, and at the same time, this method made students learn the steps of the nursing process, particularly in nursing diagnoses, and apply them in the clinic.

One limitation observed was the short internship period of six days which exposed the students to a very limited number of oncological cases. Also, patients used for the case studies in this intervention were predominantly haematological cancer patients. Hence, future intervention should explore the use of patients with non-haematological illnesses, to give students a broader approach to critical thinking and clinical reasoning in this regard. The cognitive learning test designed by one of the authors would need further studies to support its metric qualities, including indices of discrimination or difficulty of test items. In short, more metric evidence would be needed. Another limitation of the study was that it only included nursing students in fourth year and the results of the study cannot be generalized to students of lower years. It is suggested that in future studies, this educational method be used with lower-semester students so that the student becomes familiar with the case method and the nursing process. Doing so, in later semesters, they will be able to diagnose the patient’s clinical problems with greater skill and develop an effective care plan.

## Conclusions

The findings of this study showed that the integrated teaching-learning method was effective in increasing cognitive learning and clinical decision-making ability of the students. Nursing educators can use this method to improve students’ cognitive and meta-cognitive skills, thereby improving nursing care quality. This means that the clinical teachers might use one or more teaching styles to ensure student learning. In fact, it must be said that clinical education is student centered, and cooperation between students and teachers is the essential part of clinical education. It is recommended to use different capacities of teaching methods according to situation, skill (course content) and learner level, especially in the field of clinical education. This study can guide nurse educators to know how to integrate case study and the nursing process in clinical settings. Further studies on the integration of the nursing process with other educational methods such as concept mapping and simulation are suggested.

## Supplementary Information

**Additional file 1.** The outline of the Integrated teaching-learning (Nursing Process and Case Study) program

## Data Availability

The datasets used during the current study are available from the corresponding author on reasonable request.

## Abbreviations

ANCOVA: Analysis of covariance
CDMNS: Clinical Decision Making in Nursing Scale
GPA: Grade Point Average
TOS: Table of Specification

## References

[1] Rassouli M, Zagheri Tafreshi M, Esmaeil M (2014). Challenges in clinical nursing education in Iran and strategies. J clin excell.

[2] Sedaghati M, Ezadi A (2014). Effectiveness of reflection in clinical education based on nursing students, perspective in Islamic Azad University- Tonecabon in 2013. Nurs Midwifery J.

[3] Fiset VJ, Graham ID, Davies BL (2017). Evidence-based practice in clinical nursing education: A scoping review. J Nurs Educ.

[4] Ghojazadeh M, Aghaei MH, Naghavi-Behzad M, Piri R, Hazrati H, Azami-Aghdash S (2014). Using concept maps for nursing education in Iran: A systematic review. Res Development Med Educ.

[5] Sayyah M, Shirbandi K, Saki-Malehi A, Rahim F (2017). Use of a problem-based learning teaching model for undergraduate medical and nursing education: a systematic review and meta-analysis. Adv Med Educ Prac.

[6] Zhang J, Cui Q (2018). Collaborative learning in higher nursing education: A systematic review. J Prof Nurs.

[7] Wang L, Chen H, Yang L, Qian C, Sun D, Sun Y (2020). Systematic training program for nursing home staff based on the concept of combination of medicine and care. Med.

[8] McPherson C, MacDonald C (2017). Blending simulation-based learning and interpretative pedagogy for undergraduate leadership competency development. J Nurs Educ.

[9] Canlas IP, Karpudewan M (2020). Blending the principles of participatory action research approach and elements of grounded theory in a disaster risk reduction education case study. Int J Qual Methods.

[10] Pu D, Ni J, Song D, Zhang W, Wang Y, Wu L, Wang X, Wang Y (2019). Influence of critical thinking disposition on the learning efficiency of problem-based learning in undergraduate medical students. BMC Med Educ.

[11] Amini A, Bayat R, Amini K (2020). Barriers to clinical education from the perspective of nursing students in Iran: an integrative review. Archiv Pharm Pract.

[12] Jamshidi N, Molazem Z, Sharif F, Torabizadeh C, Najafi Kalyani M (2016). The challenges of nursing students in the clinical learning environment: A qualitative study. Sci World J.

[13] Farzi S, Shahriari M, Farzi S (2018). Exploring the challenges of clinical education in nursing and strategies to improve it: A qualitative study. J Educ Health Promot.

[14] Heidari MR, Norouzadeh R (2015). Nursing students’ perspectives on clinical education. J Adv Med Educ Prof.

[15] Jenkins HM (1985). Improving clinical decision making in nursing. Nurse Educ.

[16] Lotfi M, Khani H, Fathi AE, Mokhtari M (2011). Effect of compound education simulation and critical thinking strategies on clinical decision making in surgical technologist students. Nurs midwifery J.

[17] Hartell E, Strimel GJ (2019). What is it called and how does it work: examining content validity and item design of teacher-made tests. Int J  Tech Des Educ.

[18] Musah MB, Al-Hudawi S, Tahir LM, Kamil M (2015). Validity of teacher-made assessment: A table of specification approach. Asian Soc Sci.

[19] Fives H, DiDonato-Barnes N (2013). Classroom test construction: The power of a table of specifications. Pract Assess Res Eval.

[20] Karimi-Moonaghi H, Dabbaghi F, Oskouie Seid F, Vehviläinen-Julkunen K, Binaghi T (2010). Teaching style in clinical nursing education: A qualitative study of Iranian nursing teachers’ experiences. Nurse educ pract.

[21] Jeppesen KH, Christiansen S, Frederiksen K (2017). Education of student nurses–A systematic literature review. Nurse Educ Today.

[22] Tseng H-C, Chou F-H, Wang H-H, Ko H-K, Jian S-Y, Weng W-C (2011). The effectiveness of problem-based learning and concept mapping among Taiwanese registered nursing students. Nurse Educ Today.

[23] Wang J-J, Lo C-HK, Ku Y-L (2004). Problem solving strategies integrated into nursing process to promote clinical problem solving abilities of RN-BSN students. Nurse Educ Today.

[24] Rowe M, Frantz J, Bozalek V (2012). The role of blended learning in the clinical education of healthcare students: a systematic review. Med Teach.

[25] Hinneburg J, Hecht  L, Berger-Höger B, Buhse S, Lühnen J, Steckelberg A (2020). Development and piloting of a blended learning training programme for physicians and medical students to enhance their competences in evidence-based decision-making.. Zeitschrift für Evidenz, Fortbildung und Qualität im Gesundheitswesen.

[26] Parandavar N, Rezaee R, Mosallanejad L, Mosallanejad Z (2019). Designing a blended training program and its effects on clinical practice and clinical reasoning in midwifery students. J Educ Health Promot.

[27] Alfayoumi I (2019). The impact of combining concept-based learning and concept-mapping pedagogies on nursing students’ clinical reasoning abilities. Nurse Educ Today.

[28] Shahsavari Isfahani S. Designing and implementing the integrated learning program in nursing education: The integration of problem-based learning and role playing methods in teaching the practical part of patient education. J Med Cultivation. 2017;26(3):219–27.

