# Supplementary material for: Impact of integrated teaching-learning method on oncology clinical decision-making ability and cognitive learning of nursing students
Source: BMC Med Educ. 2022 Feb 19;22:107. doi: 10.1186/s12909-022-03168-2 (PMC8857849; doi:10.1186/s12909-022-03168-2)
Supplement: Supplementary file 1 — Additional file 1. The outline of the Integrated teaching-learning (Nursing Process and Case Study) program [file 12909_2022_3168_MOESM1_ESM.docx]

| **Additional file 1. The outline of the Integrated teaching-learning (Nursing Process and Case Study) program** | |
| --- | --- |
| **Session Educational content for the intervention group** | |
| **First day of internship** | ⯀Explaining the Integrated teaching-learning program for students of the intervention group.  ⯀Selecting of the oncology and hematology cases for students (one case per student).  ⯀Selecting the cases based on the students learning needs and interest.  ⯀Study of selected cases by the students and preparation for assessment. |
| **Second day of internship** | ⯀Recording a brief and accurate description all of patients events and issues that patients encountered during the hospitalization period and using all such information to create scenarios.  ⯀The following activities were required of the students and instructor in order to set the scenario:  1. Patient's social and personal information, major problems, reason for visiting a physician, medication history, method of transfer to the emergency room, and initial emergency room measures (this information was obtained in an interview with the patients and from a review of their medical record).  2. History of the disease, risk factors, lifestyle, family history, physical examinations, review of various body systems, and interpretation of blood tests.  ⯀Having the instructor guide the students through the case studies and answer their questions (particularly regarding the interpretation of the test results and the chemotherapy regimen)  ⯀Designing the appropriate questions about the case (for each case, 8 to 10 questions), after creation of the scenario.  ⯀Guide the students using the problem-solving method and introduce them to various resources to get answers to the questions and complete information about the case. |
| **Third day of internship** | ⯀Analysis of the patient’s information based on the scenario and assessment forms.(The students compared the information obtained from the medical records to the information obtained from the case)  ⯀Creation of a list of patient’s problems after analyzing the data. (All of the patient's issues were considered, including physical, psychological, social, economic, lifestyle, and risk factors)  ⯀ Giving guidance to the students and providing necessary feedback for analyzing information and extracting clinical problems.  ⯀Ensuring the documentation of the appropriate nursing diagnosis by the students and prioritizing them based on the needs and problems of the patients.  ⯀ Writing the nursing intervention related to the nursing diagnosis and developing a patient care plan.  ⯀Educating patients on some of these intervention and assisting them in carrying them out, prior to discharge. |
| **Fourth, fifth and sixth day** | ⯀Presentation of cases during a group session with the instructor present ( two of the case study were presented each day by the students in the group in the conference hall of the ward)  ⯀ The instructor asks 8 to 10 questions about cases and allowing other students in the group express their opinions.  ⯀Discussing the nursing diagnosis and care plane in the group.  ⯀Providing the necessary feedback to achieve the learning goals and care plan. |
